# 

**Published:** 1868-10-01

**Authors:** 


### *CONTENTS:*

ORIGINAL PAPERS:

Pathogenesis. The Pathological Anatomy and Pathogenesis of Disseminated Chronic Pneumonia, and of Pulmonary Tubercles. Translated by Walter Hay, M.D., Associate Editor, Chicago. Concluded from page 597 .......... 615

Elixirs of Calisaya and Calisaya and Iron .......... 625

A Case of Apoplexy .......... 628

Fluid Extracts .......... 635

Prolonged Retention of Life by Infants who have not Breathed.... 638

Additional Remarks on an Obstetrical Case .......... 639

PHILADELPHIA CORRESPONDENCE:

Uterine Diseases .......... 640

BOOKS RECEIVED .......... 643

EDITORIAL:

Medical College Fees. Free Medical Education .......... 645

Responsibility .......... 646

